# Supplementary figures and images for: A neurotransmitter atlas of C. elegans males and hermaphrodites
Source: bioRxiv. 2024 Jun 7:2023.12.24.573258. Preprint. [Version 2] doi: 10.1101/2023.12.24.573258 (PMC11185579; doi:10.1101/2023.12.24.573258)

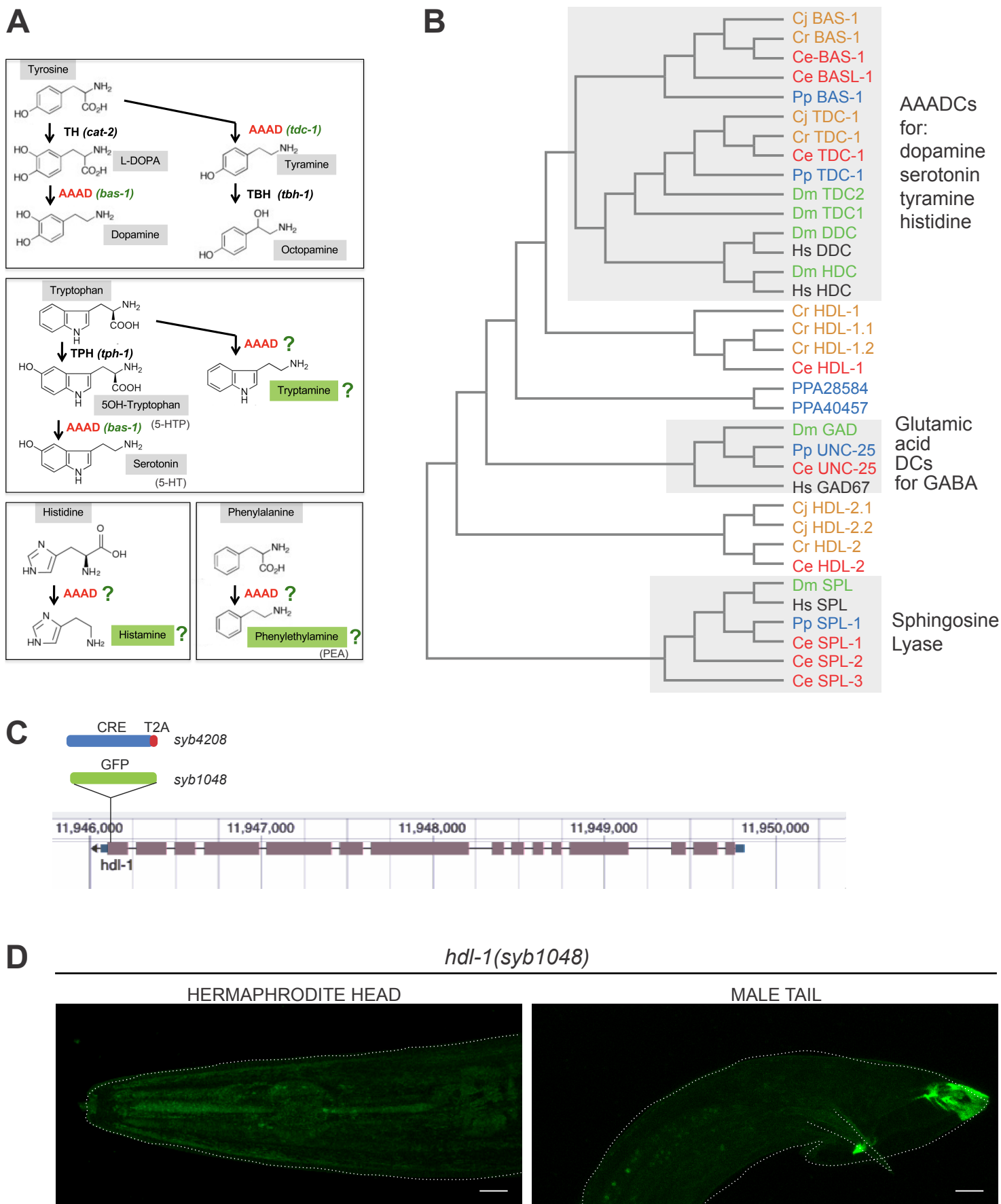

FIG. S1

Supplement: Supplement 1 — Figure S1. Use of AAADs (Aromatic Amino Acid Decarboxylases) in C. elegans. (A) Biosynthesis of biogenic amines involve the use of AAADs. Modified from (Hobert 2013) (B) Phylogenetic trees of amino acid decarboxylases. The only AAAD that displays reasonable sequence similarity to neurotransmitter-producing AAADs is the hdl-1 gene (Hare and Loer 2004; Hobert 2013). (C, D) We engineered a GFP reporter allele for hdl-1 (syb1048) (C) and did not detect any expression (D). We also attempted but failed at amplifying weak expression signals by using a Cre recombination strategy (C, syb4208, see Methods). [file media-1.pdf]

# A

original GFP+DIC images

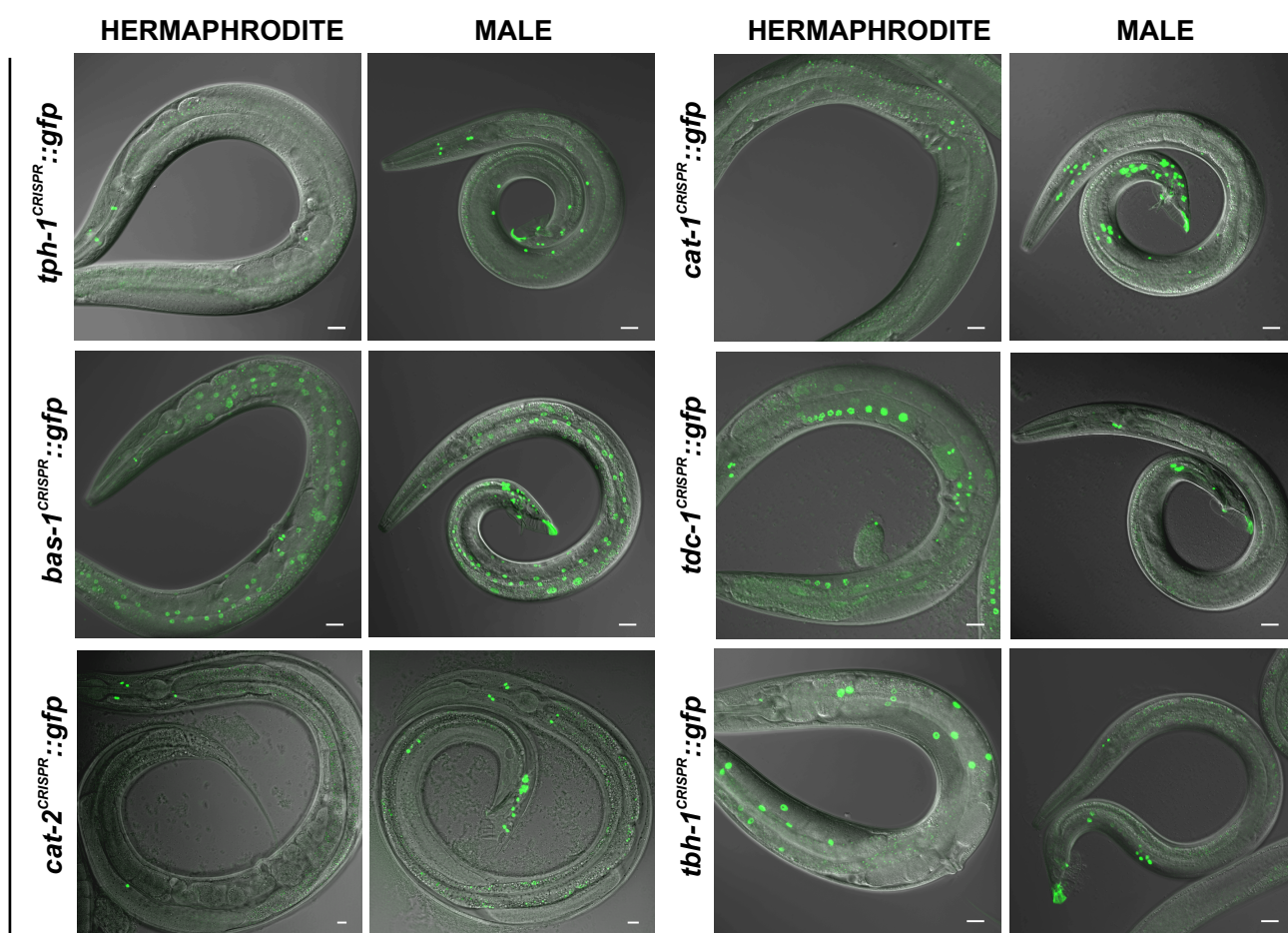

# B

grayscale images (for clearer labeling)

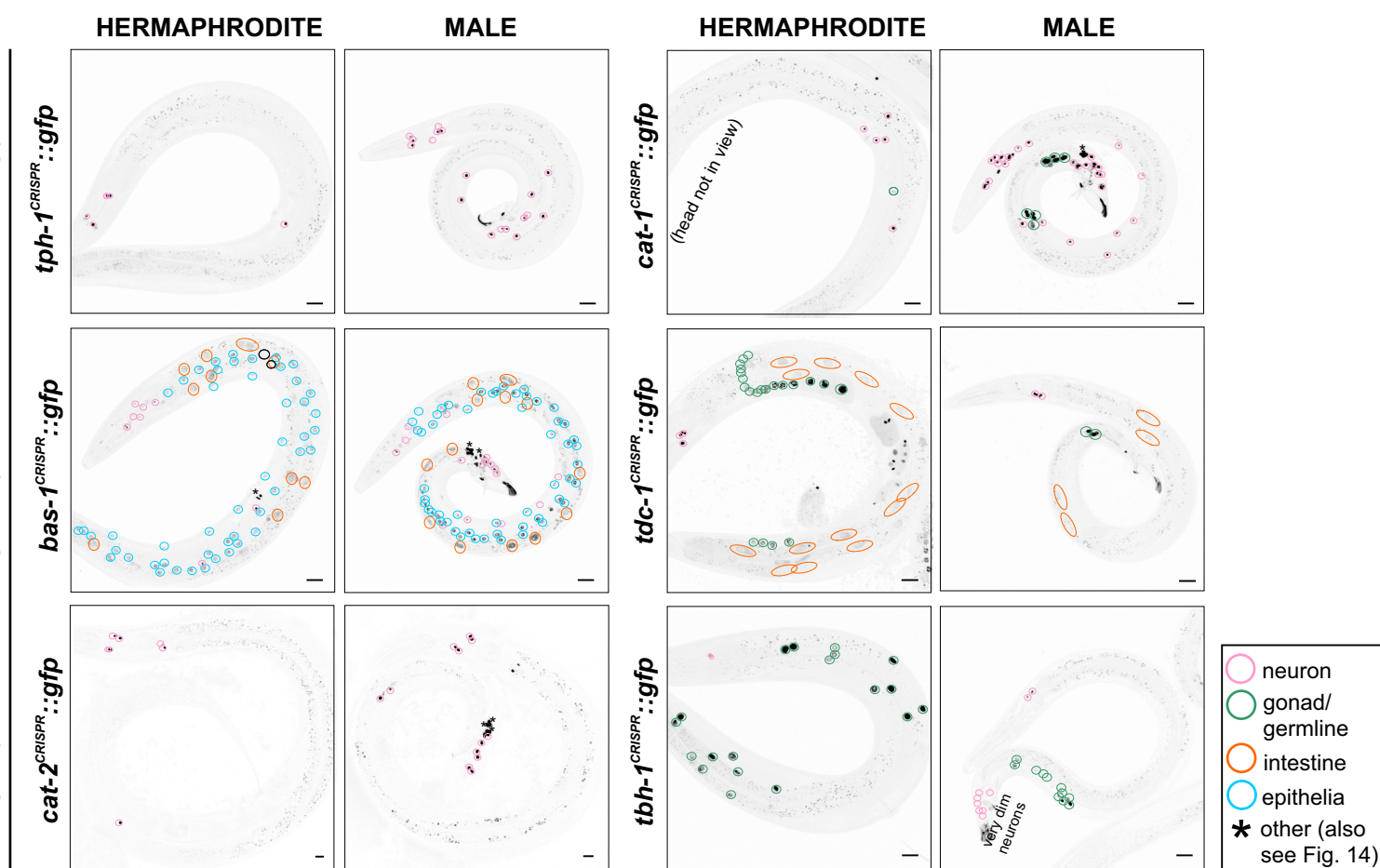

FIG. S3

Supplement: Supplement 3 — Figure S3. Whole-worm images showing monoaminergic pathway gene expression in different tissue types. Monoaminergic neurotransmitter reporters show abundant expression outside of the nervous system. Lateral views of entire worms expressing the tph-1/TPH (syb6451), bas-1/AAAD (syb5923), cat-2/TH (syb8255), cat-1/VMAT (syb6486), tdc-1/TDC (syb7768), and tbh-1/TBH (syb7786) reporter alleles. (A) GFP and DIC views. (B) Grayscale views of the GFP signal with tissue types labeled as noted on the figure. Scale bars, 20 μm. For more details, see Fig. 14. [file media-3.pdf]
