# Supplementary material for: A neurotransmitter atlas of C. elegans males and hermaphrodites
Source: bioRxiv. 2024 Jun 7:2023.12.24.573258. Preprint. [Version 2] doi: 10.1101/2023.12.24.573258 (PMC11185579; doi:10.1101/2023.12.24.573258)
Supplement: Supplement 2 — Figure S2. tph-1/TPH reporter allele expression in the hermaphrodite larvae. Hermaphrodite heads from different larval stages (L1 to L4) and young adults expressing tph-1(syb6451). tph-1 expression in the NSML/R and ADFL/R neuron pairs and in the MI neuron was visible across all larval stages and during adulthood. MI expression was validated using otIs518[eat-4(fosmid)::SL2::mCherry::H2B], a reporter for the glutamatergic identity of MI. Non-neuronal expression of tph-1 (asterisks) could be detected in a subset of pharyngeal muscles in the L1 to L4 larval stages but very dim or no expression was detected in young adults. Scale bars, 10 μm. [file media-2.pdf]

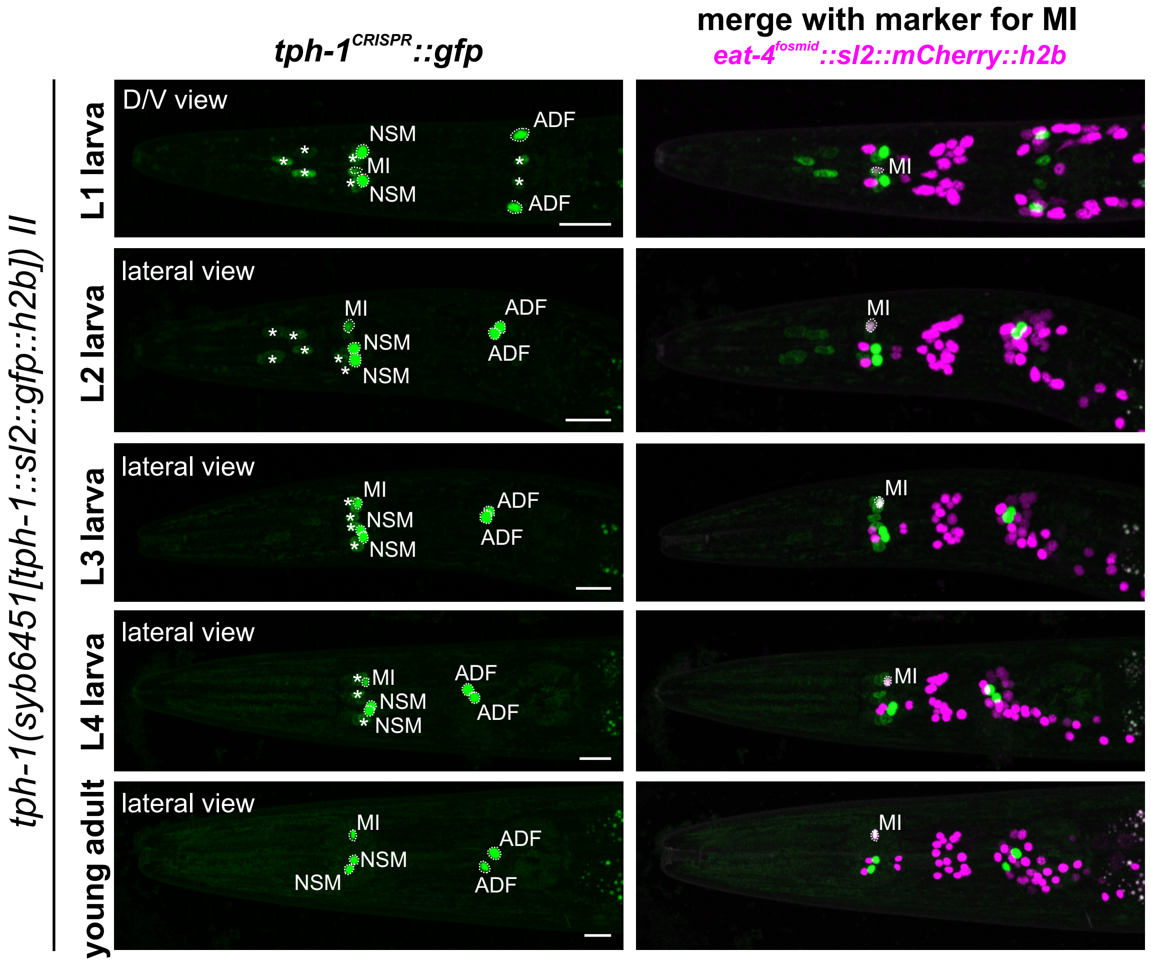

tph-1^CRISPR::gfp
merge with marker for MI
eat-4^fosmid::sl2::mCherry::h2b
tph-1(syb6451[tph-1::sl2::gfp::h2b]) II
L1 larva
D/V view
NSM
MI
NSM
ADF
ADF
L2 larva
lateral view
MI
NSM
NSM
ADF
ADF
L3 larva
lateral view
MI
NSM
NSM
ADF
ADF
L4 larva
lateral view
MI
NSM
NSM
ADF
ADF
young adult
lateral view
MI
NSM
NSM
ADF
ADF

FIG. S2
